# Mandibular osteomyelitis in South American camelids without primary dental pathologies (4 cases)

**DOI:** 10.1186/s12917-026-05768-2

**Published:** 2026-07-30

**Authors:** Nora Mareike Biermann, Thomas Wittek, Cassandra Eibl, Eva-Maria Bartl, Maria Prüllage

**Affiliations:** 1 Clinical Centre of Equine Health and Research, Clinical Department for Companion Animals and Horses, University of Veterinary Medicine Vienna, Vienna, Austria; 2 Clinical Centre for Ruminants, Camelids and Herd Health Management, Clinical Department for Farm Animals and Food System Transformation, Thomas Wittek, University of Veterinary Medicine Vienna, Vienna, Austria; 3 Unit of Diagnostic Imaging, Clinical Department for Companion Animals and Horses, University of Veterinary Medicine Vienna, Vienna, Austria

**Keywords:** Alpacas, Lamas, Apical infection, Camelid surgery, Lower jaw, Bone infection

## Abstract

**Background:**

Mandibular osteomyelitis is a clinically relevant condition in South American camelids (SACs). It is most commonly reported secondary to dental disease. Information on primary mandibular osteomyelitis without dental involvement remain scarce in these species. This case series reports the clinical presentation, diagnostic findings, treatment, and outcome of four SACs diagnosed with severe mandibular osteomyelitis in the absence of primary dental disease.

**Case presentation:**

Four SACs (three alpacas and one llama) presented with either unilateral or bilateral mandibular swelling and ventral draining tracts. Oral examination revealed mandibular bone thickening and purulent fistulae in close proximity to the cheek teeth, however, none of the teeth showed clinical abnormalities, like purulent discharge from their gingival sulci or abnormal mobility upon oral examination.

Computed tomography (CT) was performed in all cases and demonstrated extensive osteolytic lesions of the mandibular body, marked periosteal reaction, multiple osseous sequestra, and varying degrees of surrounding soft tissue swelling. CT-findings did not indicate primary dental involvement like signs of apical infection, although some periodontal changes could be observed.

Treatment primarily consisted of aggressive surgical debridement, removal of sequestrated bone without dental extraction, and short courses of systemic antimicrobial and anti-inflammatory therapy.Repeated follow-up examinations enabled further debridement when necessary and promoted progressive healing. Clinical outcomes were favorable, with long-term resolution of clinical signs reported in most animals while preserving clinically normal teeth.

**Conclusions:**

This case series highlights that severe mandibular osteomyelitis can occur independently of dental disease in SACs. Computed tomography proved essential for accurate diagnosis and surgical planning, and successful treatment relied mainly on thorough surgical debridement rather than prolonged antimicrobial therapy.

These findings emphasize the importance of advanced imaging and proper follow-up care when mandibular osteomyelitis is present in these animals. Preservation of nearly all clinically healthy teeth was possible in these cases and may be advantageous for maintaining long-term masticatory function. Further research is required to elucidate underlying pathogenesis and identify potential preventative measures.

**Supplementary Information:**

The online version contains supplementary material available at https://doi.org/10.1186/s12917-026-05768-2.

## Background

Domesticated South American camelids (SACs; alpacas and llamas) are gaining increasing popularity worldwide, not only for fiber production but also as companion animals, for tourism, and in animal-assisted interventions or therapy. This is leading to increased demand of specialized veterinary care for these animals in recent years. At the same time SACs tend to conceal clinical signs of illness for long periods of time, often leading to delayed diagnoses and advanced disease stage when presented to a veterinarian [1, 2]. One condition causing mandibular swelling in SACs is osteomyelitis, an infection of the bone which, particularly in chronic disease, is often associated with marked destruction and remodeling of bony structures as well as osseous sequestrum formation [3, 4]. Both, primary and secondary osteomyelitis of the mandibular bone has been reported [5]. Secondary mandibular osteomyelitis caused by advanced dental disease appears to be the most frequent clinical presentation [3, 4, 6, 7]. To date, only a small number of cases of primary mandibular osteomyelitis have been reported in the literature and no common causes have been proposed [8].

A type of primary osteomyelitis described in cattle is “lumpy jaw” which is caused by *Actinomyces bovis* via mucosal trauma without an overt dental focus [9]. Although infections with *A. bovis* seem very uncommon in SACs [10, 11], similar routes of bacterial infection, through microtrauma or mucosal irritation, may be plausible. Feeding coarse or abrasive forage has been proposed to contribute to the development of minor mucosal lesions, serving as entry points for opportunistic bacteria [10]. Alternatively, other local or systemic factors may facilitate hematogenous spread or local seeding of pathogens. However, to date very little is known about the etiology of primary osteomyelitis in SACs.

Clinical signs of mandibular osteomyelitis in SACs are often nonspecific. They may include firm or painful mandibular swellings and, in some cases, fistula tracts. Generalized clinical signs, such as pyrexia, lethargy, or decreased appetite, are infrequently observed [4, 12]. Instead, affected animals may show only subtle changes, such as gradual weight loss, which are often overlooked [4, 12]. Hence, accurate diagnosis relies heavily on careful physical examination and appropriate imaging modalities. Conventional radiography remains the initial imaging tool of choice for evaluation of suspected bony or dental abnormalities. Radiographic findings typical for osteomyelitis include cortical lysis, periosteal reaction and bony sequestra [4]. An inherent limitation to the sensitivity of skull radiography is superimposition, and computed tomography (CT) has been shown to be superior for a correct and detailed diagnosis in other species [13, 14]. Computed tomography is offering significantly enhanced contrast resolution and the ability to review the structures in multiplanar reconstruction [15]. While there have been descriptions of normal anatomy [15, 16], CT findings in SACs with mandibular osteomyelitis, especially in cases without apparent dental disease, remain scarce in the literature.

Treatment of osteomyelitis in SACs is complex. Although prolonged systemic antimicrobial therapy has been described previously [5], the avascular nature of infected necrotic bone (sequestra) often renders systemic antibiotics alone insufficient, particularly in chronic cases [8].In cases where sequestration, abscessation, or extensive bone destruction is evident, surgical intervention is frequently required but little information on specific treatments has been published [8]. In cases of secondary osteomyelitis due to dental pathologies high recurrence rates of up to 70% have been reported [4], especially associated with incomplete removal of infected bone, re-infection via lesions communicating with the oral cavity, and delayed initiation of treatment [4]. Treatment of dental diseases in many species, including SACs, continues to advance improving the prognosis for secondary dental mandibular osteomyelitis. In cases of primary mandibular osteomyelitis, little information on treatment and prognosis is currently available although it has been proposed that prognosis may not be as favorable [8].

The objective of this case series is to describe clinical presentation, diagnostic findings, treatment and outcome of SACs with severe mandibular osteomyelitis without primary dental pathologies.

## Case 1

**Fig. 1:**
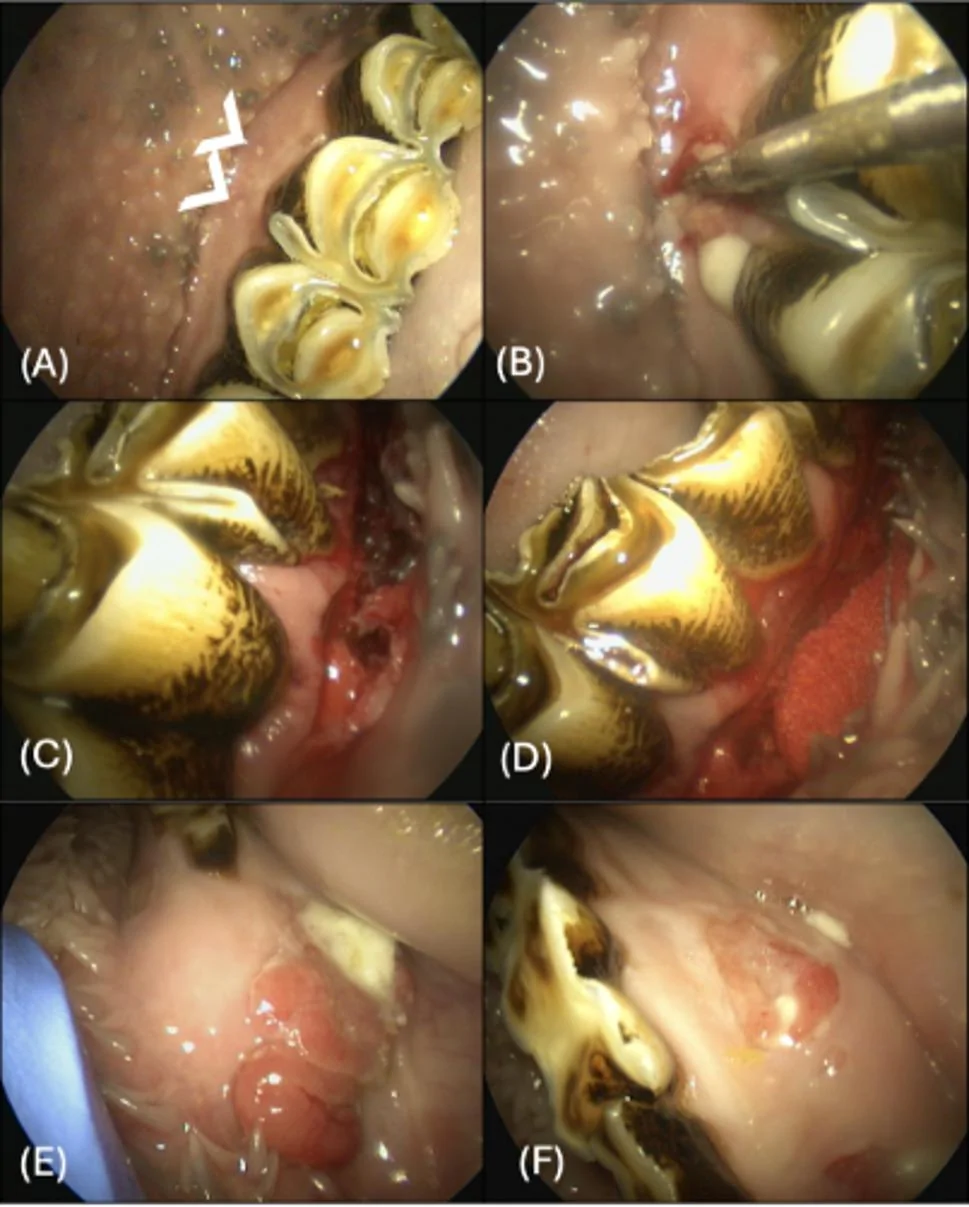
**A-F** Intraoral endoscopic view of lesions prior and after treatment of severe osteomyelitis of South American Camelids without primary dental involvement. **A** Focal mucosal hyperemia and pin-point mucosal lesions buccal to the dental arcade (open white arrows) in an alpaca with severe osteomyelitis without dental involvement at the level of M2/M3 (case 1). **B** Insertion of a dental probe into the lesions resulted in expression of purulent material, the underlying bone appeared softened (case 1). **C** After debridement of a buccally located intraoral osteomyelitis lesion (case 2). **D** The same lesion after placement of dental packing to prevent food contamination (case 2). **E** Intraoral view of granulations tissue and large amounts of purulent material rostral to P4 with severe osteomyelitis (case 3). **F** Extension of mucosal lesions at the level of M2 (case 3)

A 7-year-old female alpaca weighing 61 kg presented with a 9-month history of right mandibular swelling without dysphagia or weight loss. Based on radiographic findings the primary veterinarian started treatment with anti-inflammatories and antibiotics which failed to improve clinical signs, and the alpaca was referred eight months later for further evaluation. On presentation, vital parameters and bloodwork were within normal limits. The alpaca was premedicated with ketamine (3 mg/kg IM; Ketamidor 100 mg/mL, VetViva Richter Pharma, Austria), xylazine (0.4 mg/kg IM; Sedaxylan 20 mg/mL, Dechra, Germany), and butorphanol (0.1 mg/kg IM; Butomidor 10 mg/mL, VetViva Richter Pharma, Austria). Following sedation, a venous auricular catheter (18–22G and 22G, respectively; Vasofix Safety, Braun, Germany) was placed. Anesthesia was induced with ketamine (3 mg/kg IV) and maintained with isoflurane (Fresenius Kabi, Canada) in oxygen via orotracheal intubation (Rüschelite, Teleflex, Ireland), with spontaneous ventilation (detailed description of the anesthetic protocol can be found in supplemental information 1). An oral examination and a plain head CT (130 kV, 400 mAs, 0.8 mm, pitch 0.6; Somatom^®^ X-Cite, Siemens Healthcare, Erlangen, Germany) were performed in sternal recumbency under general anesthesia. On oral examination, the mandibular bone buccal to the right dental arcade was markedly thickened. The gingiva showed focal hyperemia and pinpoint size lesions where purulent exudate could be expressed manually (Fig. 1A). A dental probe (19’’, 1 mm tip, Promax Equine Dental, US) could be inserted up to two-centimeter into these draining tracks and the underlying bone appeared softened (Fig. 1B), dental probing of the gingival sulcus and spaces between adjacent teeth was unremarkable. All teeth were visually and on palpation clinically normal. CT imaging revealed osteomyelitis centered on the lateral aspect of the mid right mandibular body (8.1 cm in length) with minor periodontal changes of M2 and M3, but without apical involvement. Additionally, multiple isolated osseous fragments surrounded by lytic rims (involucrum) were located within the mandibular body. The adjacent soft tissues were mildly thickened and heterogeneous (Table 1). Systemic therapy with carprofen (3 mg/kg, SC, q48 h; Carprodolor 50 mg/mL, Dechra, Netherlands) and amoxicillin (15 mg/kg, IM, q48 h; Vetrimoxin 150 mg/mL, Ceva, France) was initiated immediately prior to surgery and continued for four and six days, respectively. Surgical treatment under general anesthesia included debridement of the buccal surface of the mandibular body and removal of one large sequestrum via an oral approach after placement of a modified intraoral inferior alveolar nerve block with 2 ml of 2% procaine hydrochloride (Procamidor 20 mg/mL, VetViva Richter, Austria) [17, 18]. First, the abnormal looking gingiva was incised over the length of the bony lesion using a #10 scalpel blade (Heinz Herenz Hamburg, Germany) followed by removal of necrotic bone and purulent material with a sharp 90 degree angled dental elevator (GerVetUSA, US) until only firm bone remained. Bacteriological culture was performed from purulent material but showed no growth. The site was lavaged with Ringer’s solution (B.Braun, Germany) and filled with dental packing material (Telaprep No. 1–3, Hartmann, Germany) to prevent food packing. A ventral soft tissue abscess at the level of the distal root of M2 was then opened externally using a #10 scalpel and Metzenbaum scissors following sterile preparation and debrided with a curette before lavage and bandage application. The alpaca was extubated once swallowing reflexes returned and recovered with the head maintained in a slightly elevated position to minimize the risk of aspiration. Recovery from anesthesia was uneventful and the alpaca resumed eating within hours. The bandage was changed two days later revealing a clean and dry surgical site with mild swelling. Nine days post-operatively, oral re-examination under sedation showed that the packing material was no longer in place and the defect was nearly completely granulated, with a small (2 × 2 mm) area of exposed alveolar bone at the distal extend. No more packing was required. The external site had healed, and bandaging was discontinued. The alpaca was discharged the following day with instructions to monitor for swelling, heat, pain, or reduced feed intake. Telephone follow-up two months after discharge revealed a gradual decrease in mandibular swelling. Two years after discharge the animal was doing well with complete resolution of clinical signs.

**Table 1:** Computed tomographic imaging findings of 4 South American Camelids (SACs) with severe mandibular osteomyelitis without a primary dental origin prior to treatment

CT features	Case 1	Case 2	Case 3	Case 4
Lesion location	Lateral mid 2/3 of right mandibular body (8.1 cm)	Lateral mid 2/3 of left mandibular body (12 cm)	Entire right mandibular body (17 cm) and rostral 2/3 of medial left mandibular body (10 cm)	Lateral caudal 2/3 of right mandibular body (12 cm)
Lesion center	Right mandibular body; distal M2–mesial M3	Left mandibular body; M2–M3	Right mandibular body; canine–P4 diastema	Right mandibular body; P4–M2
Soft tissue changes	Marked focal, mildly heterogeneous soft tissue thickening centred on the lesion (abscessation)	Moderate-marked extensive soft tissue swelling with small ventrolateral mineralizations	Moderate-marked extensive soft tissue swelling with gas pockets centred on the lesion (abscessation)	Moderate extensive soft tissue swelling (abscessation)
New bone formation / periosteal reaction	Solid, irregular periosteal reaction (up to 1.6 cm thick)	Sunburst-like and amorphous periosteal reaction (up to 1.3 cm thick)	Amorphous onion bulb-like new bone formation centred on the lesion; surrounding sunburst-like periosteal reaction affecting the right mandibular body and rostral medial/ventral left mandibular body	Sunburst-like periosteal reaction (up to 1.4 cm thick)
Bone lysis of mandible	Focal complete lysis of the lateral cortical bone	Focal complete lysis of the lateral cortical bone	Focal complete lysis of the right rostral mandibular body at the diastema with step formation between rostral and caudal segments; retained bone fragments within soft tissues; cortical thinning and lysis	Lysis and thinning of the lateral cortical bone
Sequestrum formation	≥ 3 partially confluent sequestra communicating with abaxial soft tissues via multiple cloacae	Multiple confluent sequestrum-like lesions	Multiple sequestra involving the left and right mandibular body (medial and lateral), symphysis and pars incisiva	Multiple sequestra along the lateral aspect of the right mandibular body
Transition zone	Long	Long	Long	Intermediate
Contact zone to adjacent teeth	Buccal alveolar bone lysis: distal M2, mesial M3; mild periodontal space widening: distal M2 and M3 roots; no apical involvement	Multifocal buccal alveolar bone lysis: M1–M2; alveolar bone thinning and irregularity: M3 roots; mild periodontal space widening: M1–M3; no apical involvement	Right: buccal and lingual alveolar bone lysis/thinning: P4–M3; mild periodontal space widening: P4 and M1. Left: distal P4 root fracture; periodontal space widening: distal and mesial P4 roots, M1 and mesial M2 root; no apical involvement	Buccal alveolar bone lysis and root thinning: P4, M1, M2 and mesial M3 root; minimal apical periodontal space widening: P4, M1 and M2; no apical involvement
Involvement of adjacent anatomical structures	Right mental foramen at lesion periphery, surrounded by periosteal new bone; neurovascular bundle courses through a canal formed by the new bone	Left mental foramen surrounded by periosteal reaction; neurovascular bundle courses through a canal formed by the new bone	Complete interruption of the mandibular canal at the diastema; left mental foramen surrounded by periosteal reaction; neurovascular bundle courses through a canal formed by the new bone	Focal discontinuity of the lateral mandibular canal; right mental foramen at lesion periphery, surrounded by periosteal new bone; neurovascular bundle courses through a canal formed by the new bone
Lymph nodes (lnn)	Mild right mandibular lymphadenomegaly (0.9 cm)	Moderate-marked left mandibular lymphadenopathy with mineralization (2.0 cm) and left medial retropharyngeal lymphadenopathy	Marked right mandibular lymphadenopathy (1.7 cm) and mild right medial retropharyngeal lymphadenopathy	Marked right mandibular lymphadenopathy (2.3 cm)
Limitation	Incisor teeth non-diagnostic due to motion artifact Medial retropharyngeal Lnn not clearly delineated			Medial retropharyngeal Lnn not clearly delineated
Others	Intracranial mineralisations	Soft tissue attenuation of pneumatized petrous bone / osseous tympanic bulla Soft tissue attenuating material in both external ear canals s.c. mineralizations of the parotid gland / ln and tongue		Infundibular gas in multiple molar teeth

## Case 2

A 7-year-old 150 kg intact male llama was presented with left mandibular swelling of unknown duration. The animal showed no additional clinical abnormalities, maintained normal appetite, and had received no prior treatment. On presentation, vital parameters were within normal limits. Oral examination revealed thickened buccal alveolar bone of the left mandible with multiple pinpoint draining tracts with purulent exudate. Bacteriological culture identified moderate growth of *Escherichia coli* and *Fusobacterium necrophorum*. All teeth appeared upon visual inspection and palpation clinically unremarkable and stable. Plain head CT (130 kV, 450 mAs, 0.8 mm, pitch 0.6) revealed osteomyelitis involving the lateral aspect of the left mid mandibular body, extending 12.1 cm in length (Table 1). Multiple sequestra of different size and moderate to severe soft tissue thickening were present as well. Induction and maintenance of general anesthesia for diagnostic imaging and surgery was similar to case 1 and following an intra-oral inferior alveolar nerve block with 4 ml of 2% procaine hydrochloride, the infected bone was debrided from the mesial root of M1 up to the distal root of M3 (Figs. 1C). Debridement of infected bone and removal of 7–10 small sequestra were performed with 90 degree angled sharp dental elevators. The caudal portion of the lesion extended ventrally near the mesial root of M3, which was two-thirds exposed during debridement. As the tooth remained stable, no extraction was performed at that time. The defect was lavaged with Ringer’s solution and filled with dental packing material (Fig. 1D). Recovery from anesthesia was uneventful, and the llama resumed normal food intake within hours. Peri- and postoperative treatment included carprofen (3 mg/kg, SC, q48 h) and amoxicillin (15 mg/kg, IM, q48 h) for five days until the first packing change. At this time, the rostral portion of the lesion contained no packing material and was covered with granulation tissue. The caudal aspect still contained packing material and moderate purulent exudate. After lavage and debridement, new packing material was applied. Nine days postoperatively, re-examination revealed advanced healing and complete granulation of the defect. Lavage was repeated, and no further packing was required. The llama was discharged the following day with instructions to monitor for changes in mastication, weight loss, recurrence of mandibular swelling or draining tract formation. Regular telephone follow-up confirmed complete resolution of clinical signs, with the animal being clinically normal nearly two years after discharge.

## Case 3

**Fig. 2:**
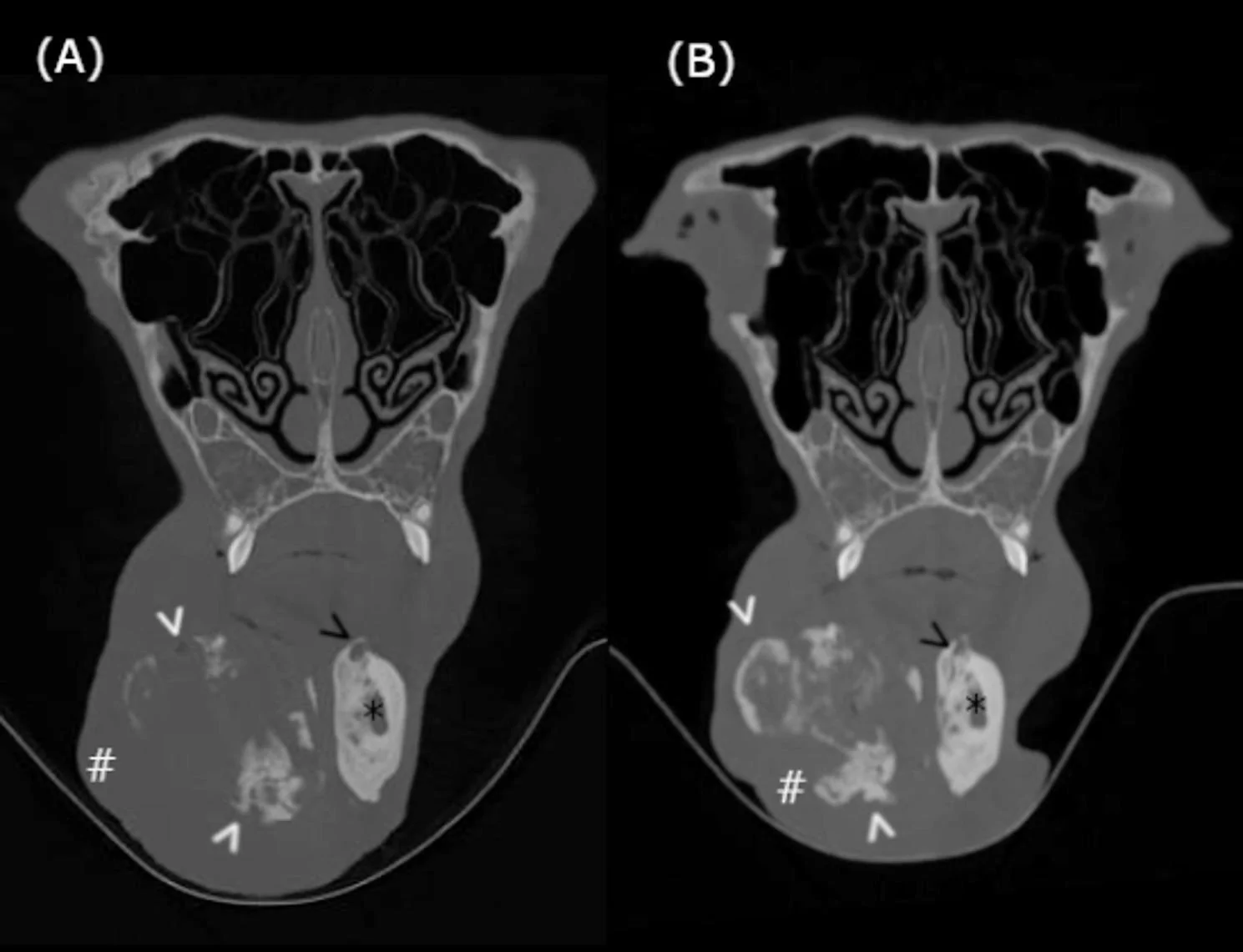
**A-B** Transverse CT images (bone window; 130 kV, 475mAs, 10 mm pitch 0.6) of Case 3 obtained prior to treatment (**A**) and one month after treatment (**B**) in case 3. Both images are acquired at approximately the same level, centered on the third maxillary premolar; the patient’s right side is displayed on the left side of the image. On the pretreatment image (**A**), marked osteolytic changes are evident in the right mandibular body, accompanied by an irregularly shaped periosteal reaction (open white arrowhead) along its contour. On the follow-up CT image (**B**), the periosteal reaction (open white arrowhead) appears smoother and more solid. The extent of osteolytic bone changes is reduced compared with the prior examination. Additionally, the associated soft tissue thickening (white hash tag) has decreased. Well-circumscribed osteolytic lesions with an adjacent solid periosteal reaction (open black arrow) are also visible along the medial aspect of the left mandibular body. The mandibular canal is indicated by a black star. White background lines indicate positioning aids

An 8.5-year-old female alpaca weighing 73.5 kg was presented with right mandibular swelling and both rostral and caudal draining tracts present for two months. No other abnormalities were reported. Physical examination and bloodwork were unremarkable aside from the mandibular lesions. Plain CT (130 kV, 475 mAs, 10 mm, pitch 0.6) was performed under general anesthesia following the same protocol (supplemental information 1) and revealed extensive osteomyelitis of the right mandible (17 cm in length) with complete discontinuity of the right mandibular body rostral to P4, and concurrent osteomyelitis of the left mandible (10.1 cm in length) (Table 1). With partially confluent lytic zones, bony sequestra were present in both mandibular bones and the markedly thickened adjacent soft tissue structures contained small gas bubbles indicating abscess or sinus tract formation. After confirming stable mandibular alignment, oral examination showed multiple draining tracts buccal and lingual to P4 (Fig. 1E), extending lingually along M1–M3 (Fig. 1F), with additional tracts and granulation tissue rostral to P4 beneath the tongue. Purulent exudate could be expressed from all areas but dental probing of the gingival sulci adjacent and in between cheek teeth revealed no abnormalities. Surgical treatment ensued after placement of an intra-oral inferior alveolar nerve block using 3 ml 2% procaine hydrochloride. Debridement with 90 degree angled sharp dental elevators removed purulent material and seven sequestra of varying sizes. During debridement, P4 became unstable and was extracted, although no direct sign of infection was observed. Debridement of the rostral lesion was performed cautiously to preserve mandibular stability. Two ventral fistula tracts were surgically approached externally after clipping and aseptic preparation. Each tract was enlarged with a #10 scalpel and Metzenbaum scissors, followed by curettage. Three sequestra were removed rostrally, creating an orocutaneous fistula, and one sequestrum was removed caudally. All sites were thoroughly lavaged using Ringer’s solution. On palpation, a thick lateral and a thinner ventral bony strut appeared to maintain mandibular stability at the time. Dental packing was placed in all defects, particularly to close the orocutaneous fistula and prevent salivary loss. A bandage was applied before recovery from anesthesia, which was uneventful. Systemic carprofen (3 mg/kg, SC, q48 h) and amoxicillin (15 mg/kg, IM, q48 h) were administered pre- and postoperatively for a total of 12 days. Due to the extensive nature of surgical intervention, the alpaca was closely monitored for discomfort but resumed eating the evening of surgery. Metamizole (60 mg/kg IM once) was given the next morning; no further analgesia was required. Bacteriological cultures from purulent material and sequestra showed no growth. Five days postoperatively, the first packing change revealed moderate purulent material at the rostral lingual defect. After topical 2% procaine hydrochloride (5 ml) and careful debridement, again multiple small sequestra were removed. Palpation confirmed stable lateral and ventral mandibular cortices. The site was lavaged and repacked. At eight days, re-examination showed early granulation particularly caudally with two additional small sequestra removed rostrally. After lavage, new packing material was placed. Twelve days postoperatively, only minimal purulent exudate remained draining from the rostral lesion, and the defect showed advanced granulation. Upon packing change three weeks after surgery, the defect was fully granulated with no visible packing material remaining, though deep placement may have allowed ingrowth into granulation tissue. Follow-up CT at four weeks confirmed this and the retained packing material was removed through a small residual ventral draining tract under sedation and local anesthesia. The CT showed reduced but persistent lytic changes, with previously extensive, sunburst-like periosteal reactions becoming more organized and solid. Soft tissue thickening and gas pockets were reduced but still present (Figs. 2). The alpaca was discharged two days later with instructions to monitor the mandible and the remaining draining tract and to return for a re-examination in six to eight weeks. Re-examination by the referring veterinarian three months after discharge revealed improved clinical signs, decreased mandibular swelling, and healing of the draining tract. No further follow-up information was available.

## Case 4

**Fig. 3:**
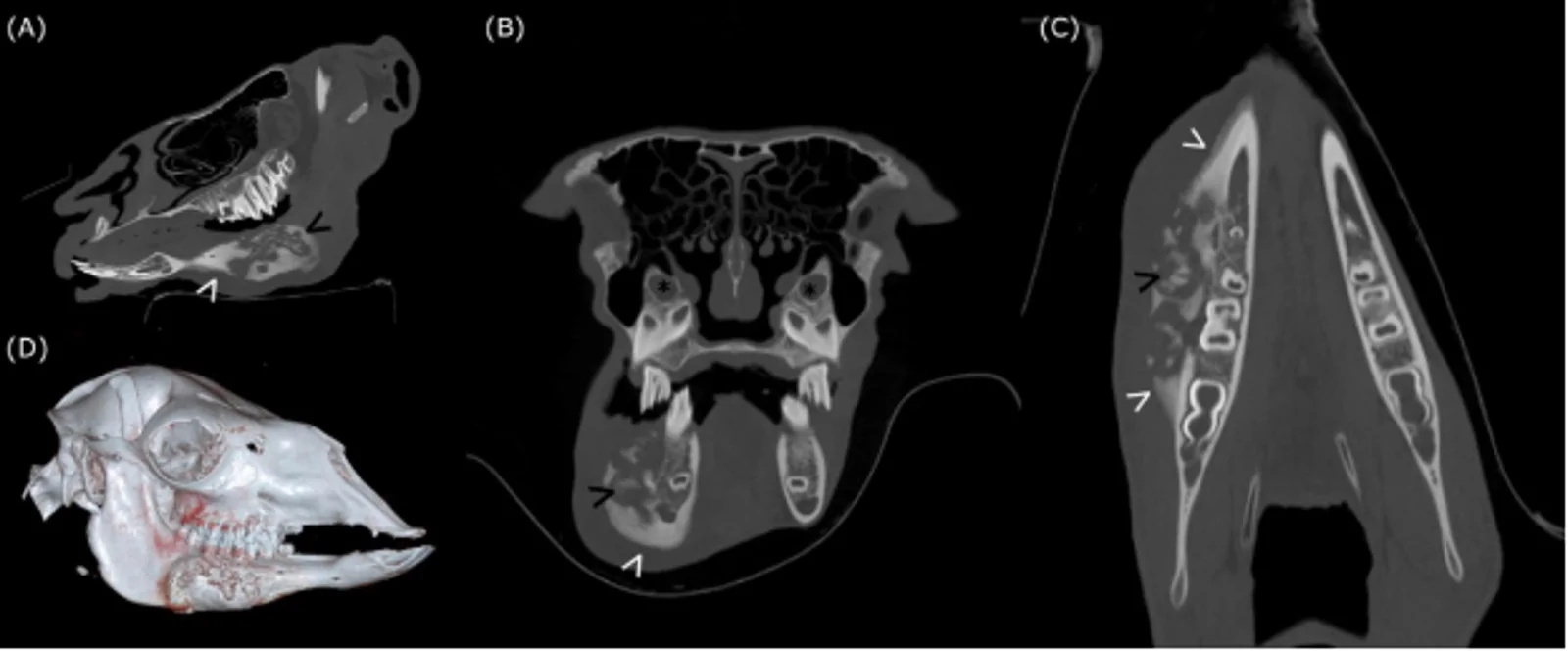
**A-D** Multiplanar reformatted CT images (bone window; 130 kV, 500 mAs, 0.8 mm, pitch 0.6) and 3D reconstruction of case 4. A monostotic aggressive bone lesion is centered on the right mandibular body. The paramedian sagittal image (**A**) is aligned with the right mandibular body; the transverse image (**B**) is at the level of the mandibular second molars; and the dorsal image (**C**) allows comparison of both mandibular bodies; the patient’s right side is displayed on the left side of the image. White background lines indicate positioning aids. The volume-rendered bone model (**D**) demonstrates the marked osseous changes from a right rostrolateral perspective. Multiple osseous fragments surrounded by a hypoattenuating rim (involucrum), consistent with bony sequestra (open black arrowhead), are present within the right mandibular body and are associated with an irregular periosteal reaction (open white arrowhead). The lesion extends to the level of the periodontal space, which otherwise appears unremarkable. In (**A**), remnants of the right maxillary deciduous second premolar overlie the permanent second premolar. In (**B**), the infraorbital canals (black asterisk) are shown for orientation

A 3-year-old female alpaca weighing 69.8 kg presented with a one-month history of right mandibular swelling. Initial physical examination and blood work were unremarkable. Plain CT (130 kV, 500 mAs, 0.8 mm, pitch 0.6) performed under general anesthesia (supplemental information 1) and revealed osteomyelitis along the buccal aspect of the caudal two-thirds of the right mandibular body (11.8 cm in length) with multiple sequestra and surrounded by moderate soft tissue thickening (Table 1; Fig. 3). Oral examination identified draining tracts with purulent exudate buccal to the right dental mandibular arcade, without dental abnormalities upon visual inspection and palpation of each tooth. Intra-oral and external debridement, lavage and packing were performed similarly to previous cases at the level of P4 up to M3. Samples of purulent material were collected for bacteriology, and the necrotic cavity of the sequestrum and the involucrum were curetted and lavaged before bandaging. Recovery from anesthesia was uneventful. Culture revealed heavy growth of *Fusobacterium necrophorum*. Systemic therapy included carprofen (3 mg/kg, SC, q48 h) and amoxicillin (15 mg/kg, IM, q48 h) for ten days. At the first packing change five days postoperatively, the rostral defect showed granulation tissue, while the deep distal part contained moderate purulent material; after debridement and lavage, new packing was applied. The external site was locally anesthetized (3 ml 2% procaine hydrochloride SQ), debrided, and rebandaged, with only minimal discharge left and firm bone palpable. Two weeks postoperatively, re-examination revealed complete granulation of the defect, epithelialization of the rostral portion, and complete healing of the soft tissue abscess. A firm, non-painful bony swelling remained. The alpaca was discharged the following day with instructions for continued monitoring. Telephone follow-up revealed complete resolution of clinical signs, and the animal remained clinically normal 1.5 years later. 

## Discussion

This case series describes four cases of severe mandibular osteomyelitis, and although lesions were in all cases in close proximity to the cheek teeth, oral examination and CT findings did not indicate direct involvement of apical dental structures.

The presented cases highlight several important features for the diagnosis and treatment of primary mandibular osteomyelitis in SACs. Similar to findings in dogs and horses [13, 14, 19], CT imaging substantially contributes to establishing an accurate diagnosis and development of a treatment plan. While systemic antibiotic therapy was used as an adjunctive therapeutic measure, the primary treatment consisted of aggressive surgical debridement, removal of sequestrated bone, and appropriate follow-up care. This suggests that extraction of cheek teeth is only necessary when there is clear evidence of apical infection or instability.

In agreement with findings in horses and other species, where CT has been shown to be superior to radiography [13, 14, 20] it also proved instrumental in defining the extent of bone involvement and its relationship to adjacent dental structures. This is due not only to the advantages of three-dimensional imaging but also to the inherent limitations of conventional radiography. Alveolar bone loss is generally not detectable radiographically until 30–50% of the bone mineral content in a specific area has been destroyed [21]. In all four cases a primarily osteolytic monostotic, aggressive bone lesion with a long transition zone of either the right or left mandibular body and both mandibles in case three was seen in CT. Consistent findings were severe periosteal reactions, moderate to marked soft tissue swellings and sequestra formation. Importantly, adjacent teeth showed widening and partial disruption of the periodontal spaces and alveolar bone. However, unlike cases of osteomyelitis secondary to dental disease, no abnormalities were identified in the apical regions of any tooth. Based on the favorable clinical outcome of these cases the preservation of teeth without apical infection points out that the apical aspect of cheek teeth appears to be of particular clinical relevance for teeth viability. This is likely due to involvement of neurovascular structures and resulting pulpitis while periapical changes mostly affect the alveolar bone and periodontium.

While there are no studies in SACs on the long-term effect of cheek tooth extraction, preservation of grinding surface in other herbivores (e.g. horses) has been shown to be essential for proper food processing in older animals [22, 23] which likely also applies to SACs. Dental extraction should therefore be carefully considered which also includes the consideration of only removing either mesial or distal roots [24] since it has been shown that particularly with increasing age separation of pulp chambers is commonly seen in SACs [15].

CT imaging also enabled precise quantification and localization of sequestra and the evaluation of new bone formation or lysis. Such detailed assessment supports surgical planning, allowing targeted debridement while preserving viable bone and uninvolved dental structures. These findings are in accordance with Po et al. (2022) [8]. The intravenous application of iodinated contrast media for improved evaluation of the soft tissue structures (e.g. abscesses, draining tracts) was not performed as determined by the European drug legislation prohibiting use of contrast media in food producing animals. Nevertheless, the evaluation of the osseous and dental changes is independent of contrast media use.

The commonly encountered chronicity of disease associated with mandibular osteomyelitis in SACs can increase the risk of pathologic fractures. Combined with a perceived high rate of reinfection, prognosis has been previously stated to be guarded [8]. Due to the high rate of recurrence of infection, prolonged courses of systemic antibiotic treatment (4–6 weeks) were advocated [5]. A recently described case of primary mandibular osteomyelitis was successfully treated with a combination of surgical debridement and use of systemic and local application of antibiotics [8]. In the present cases, treatment relied primarily on extensive debridement combined with repeated follow-up examination and a relatively short course (between 5 and 12 days) of systemic antibiotic treatment which is in line with efforts to reduce use and duration of antibiotic treatment in veterinary species [25, 26]. In our experiences, surgical removal of necrotic and sequestrated bone is the most essential treatment step, as sequestra act as foreign bodies [3] which may also hinder systemic antibiotics to act effectively. Repeat examinations in these cases are crucial to allow for repeated debridement when necessary.

Secondary osteomyelitis associated with dental disease is commonly reported [5, 12], and the absence of direct dental involvement in this study suggests that the infection may have seeded via other sources or routes. In three out of four animals, lesions were located at the lateral aspects of the body of the mandible with varying degrees of buccal involvement of alveolar bone. The similar presentation in these cases might be indicative of a common etiology, e.g. defect in the buccal mucosa; however, at the time of oral examination the only mucosal lesions identified were chronic draining tracts and inflammation. Therefore, the etiology of primary mandibular osteomyelitis in these cases remains uncertain. Regular routine oral examination at herd level would help in early disease detection and to identify underlying pathologies.

Isolation of *Fusobacterium necropherum* (in two cases in this study) has been previously described in SACs and ruminants from a range of abscesses [10, 27, 28]. This organism is a common anaerobic pathogen in the gastrointestinal tract of ruminants and SACs [5, 29]. Its isolation from mandibular osteomyelitis lesions may support the hypothesis of opportunistic infection following mucosal disruption. Since it is an anaerobic pathogen, opening of abscesses and debridement with exposure to oxygen might be one of the most effective means of treatment. In the two cases with negative culture, it may be explained by the anaerobic nature of the causative organisms and inhered difficulties with conventional culture methods. Additionally, successful culture and sensitivity of causative agents can be impaired by antibiotic treatment prior to referral. Ultimately, too little information is provided by this case series, and further investigations into bacterial pathogens causing primary mandibular osteomyelitis are warranted.

Limitations of the present case series include the small number of animals, variable disease severity, and inconsistent follow-up periods, which relied largely on owner observations. Ideally, follow-up examinations, including oral examinations and CT imaging, as well as histological examination of adjacent dental structures would better support both the absence of primary dental involvement and the favorable prognosis. Nevertheless, the consistent absence of evidence for primary involvement of dental structures across all four cases underscores the need for further investigation into non-dental origins of mandibular bone infections in SACs. Additionally, histopathological assessment of sequestered bone and debrided material might have added further information and could have contributed to a more comprehensive characterization of the lesions but was not performed in these cases.

## Conclusion

While dental disease remains a well-established cause, this case series demonstrates that severe mandibular osteomyelitis may occur in the absence of primary dental involvement. These cases showed that CT-imaging is an essential diagnostic tool for delineating lesion extent and guiding surgical management. Successful treatment relies primarily on thorough surgical debridement and repeated follow-up examination.

Further research is needed to clarify the pathogenesis of primary osteomyelitis in these species, particularly regarding the role of mucosal injury and opportunistic bacterial infection. Improved understanding of etiologic factors, supported by advanced imaging, might aid in the development of preventative management strategies.

## Supplementary Information

Supplementary Material 1.

## Data Availability

The original contributions presented in the study are included in the article, further inquiries can be directed to the corresponding author.
