# Supplementary material for: Mandibular osteomyelitis in South American camelids without primary dental pathologies (4 cases)
Source: BMC Vet Res. 2026 Jul 30;22:683. doi: 10.1186/s12917-026-05768-2 (PMC13753269; doi:10.1186/s12917-026-05768-2)
Supplement: Supplementary file 1 [file 12917_2026_5768_MOESM1_ESM.docx]

**Supplemental information**

**1. Detailed anesthetic protocol South American camelids (SACs)**

South American camelids (SACs) were premedicated with ketamine (2–3 mg/kg IM; Ketamidor 100 mg/mL, VetViva Richter Pharma, Austria), xylazine (0.2–0.5 mg/kg IM; Sedaxylan 20 mg/mL, Dechra, Germany), and butorphanol (0.1–0.3 mg/kg IM; Butomidor 10 mg/kg, VetViva, Richter Pharma, Austria). Following sedation, animals resumed sternal recumbency within 5–10 minutes, and a venous catheter (18-22G, Vasofix Safety, Braun, Germany) was placed in an auricular vein. Pulse oximetry (Rad-G, Masimo, US) was used for initial non-invasive monitoring until placement of an arterial catheter (22G, Vasofix Safety, Braun, Germany) in the auricular artery for invasive blood pressure monitoring and arterial blood sampling.

Anesthesia was induced with ketamine (2–3 mg/kg IV) and maintained with isoflurane (Fresenius Kabi, Canada) in oxygen delivered via an orotracheal tube (Rüschelite, Teleflex, Ireland). Animals breathed spontaneously throughout anesthesia. End-tidal isoflurane concentrations were maintained between 1.1% and 1.5%. In two animals, a ketamine continuous rate infusion (10 µg/kg/min IV) was administered during anesthesia and discontinued approximately 20 minutes before completion of the procedure. Lactated balanced crystalloid fluids (Vetifundin®, Oög GmbH, Austria) were administered intravenously at 5 mL/kg/h throughout anesthesia.

An intraoral mandibular nerve block was performed in all SACs using procaine hydrochloride (2–4 mL; Procamidor 20 mg/mL, VetViva Richter Pharma, Austria) to provide additional perioperative analgesia.

Throughout anesthesia, heart rate, respiratory rate, core body temperature, invasive arterial blood pressure, peripheral oxygen saturation (SpO₂), end-tidal carbon dioxide concentration (ETCO₂), end-tidal isoflurane concentration, and repeated arterial blood gas analyses were performed and monitored.

Animals were extubated once they were able to swollow spontaneously. Their heads were kept in a slightly elevated position with the nose pointing downwards to minimize the risk of saliva aspiration during recovery. Once the animals stood up they were transferred to their stalls.
